# p130Cas/*BCAR1* and p140Cap/*SRCIN1* Adaptors: The Yin Yang in Breast Cancer?

**DOI:** 10.3389/fcell.2021.729093

**Published:** 2021-10-11

**Authors:** Giorgia Centonze, Dora Natalini, Vincenzo Salemme, Andrea Costamagna, Sara Cabodi, Paola Defilippi

**Affiliations:** Department of Molecular Biotechnology and Health Sciences, Molecular Biotechnology Center, University of Torino, Turin, Italy

**Keywords:** breast cancer, cell signaling, mouse model, adaptor protein, epithelial – mesenchymal – transition, protein interactome

## Abstract

p130Cas/*BCAR1* is an adaptor protein devoid of any enzymatic or transcriptional activity, whose modular structure with various binding motifs, allows the formation of multi-protein signaling complexes. This results in the induction and/or maintenance of signaling pathways with pleiotropic effects on cell motility, cell adhesion, cytoskeleton remodeling, invasion, survival, and proliferation. Deregulation of p130Cas/*BCAR1* adaptor protein has been extensively demonstrated in a variety of human cancers in which overexpression of p130Cas/BCAR1 correlates with increased malignancy. p140Cap (p130Cas associated protein), encoded by the *SRCIN1* gene, has been discovered by affinity chromatography and mass spectrometry analysis of putative interactors of p130Cas. It came out that p140Cap associates with p130Cas not directly but through its interaction with the Src Kinase. p140Cap is highly expressed in neurons and to a lesser extent in epithelial tissues such as the mammary gland. Strikingly, *in vivo* and *in vitro* analysis identified its tumor suppressive role in breast cancer and in neuroblastoma, showing an inverse correlation between p140Cap expression in tumors and tumor progression. In this review, a synopsis of 15 years of research on the role of p130Cas/*BCAR1* and p140Cap/*SRCIN1* in breast cancer will be presented.

## p130Cas/*BCAR1* Gene and Protein

The *BCAR1* gene, the human ortholog of p130Cas, was independently identified as Breast Cancer Antiestrogen Resistance 1. *BCAR1* is localized on chromosome 16 on region q, on the negative strand and has seven exons, with multiple alternative first exons. p130Cas/BCAR1 protein (p130 Crk-associated substrate) is a member of the Cas (Crk-associated substrate) family that consists of five distinct adaptor proteins: p130Cas/BCAR1, Nedd9 (Neural precursor cell expressed, developmentally downregulated 9), Human enhancer of filamentation-1 (HEF-1 or CAs-L), EFS (Embryonal Fyn-associated substrate), and CASS4 (Cas scaffolding protein family member 4). All the family proteins are characterized by a similar modular structure with several interaction domains and multiple tyrosine and serine phosphorylation motifs (Defilippi et al., 2006; Cabodi et al., 2010; Tikhmyanova et al., 2010). The structural features of the p130Cas/BCAR1 protein includes an amino (N)-terminal Src-homology 3 (SH3) domain, an adjacent large substrate-binding domain containing 15 repetitions of the YxxP motif, a main site of tyrosine phosphorylation on the p130Cas/*BCAR1* molecule that provides SH2-binding sites, a proline and serine rich region, a C-terminal part composed by binding sites for the SH2 and SH3 domains of Src (YDYVHL and RPLPSPP, respectively) and a highly conserved four-helix bundle [Focal Adhesion Targeting (FAT) domain] (Defilippi et al., 2006; Cabodi et al., 2010; Tikhmyanova et al., 2010).

p130Cas serves as an adaptor protein in multiprotein complexes, integrating signals from the extracellular matrix environment, soluble ligands, and mechanical stress. Tyrosine phosphorylation of the substrate-binding domain by Src-family kinases enables its interaction with Crk, Crk-L, CRKII, and Nck adaptors (Schlaepfer et al., 1997). The assembly of p130Cas-Crk-dedicator of cytokinesis 1 (DOCK1; also known as DOCK180) complex allows efficient recruitment and localization of small GTPase RAC1 at the membrane. This event induces actin cytoskeleton remodeling, pseudopodia extension and focal adhesion turnover resulting in increased cell migration (Cabodi et al., 2010). The extent of this phosphorylation is regulated by the mechanical forces acting on the cell that stretch the substrate-binding domain and expose hidden tyrosines to phosphorylation (Sawada et al., 2006). The SH3 domain mediates the interaction of p130Cas with polyproline motifs of FAK, PYK2/RAFTK, and FRNK kinases, PTP1B, PTP-PEST phosphatases, and other proteins (C3G, CMS, CIZ, and Vinculin) that regulate the recruitment and/or activation of Src-family kinases (Polte and Hanks, 1995; Liu et al., 1996; Garton et al., 1997; Li and Earp, 1997; Kirsch et al., 1998, 1999; Nakamoto et al., 2000; Janostiak et al., 2014). The C-terminal domain of p130Cas is crucial to allow the binding of Src-family kinases and the targeting of p130Cas to focal adhesions (Nakamoto et al., 1997). Moreover, previous studies demonstrated the contribution of the C-terminal domain to dimer formation and association with BCAR3, NEDD9, and p140Cap (Nakamoto et al., 1997; Law et al., 1999; Di Stefano et al., 2004; Branis et al., 2017).

p130Cas is ubiquitously expressed and plays a crucial role in early mouse development. Indeed, mouse embryos in which p130Cas was knocked-out die at 12.5 for massive cardiac and circulatory dysfunction (Honda et al., 1998). Although the expression of p130Cas has been implicated in the regulation of mammary, bone, brain, muscle, and liver homeostasis (Camacho Leal Mdel et al., 2015), the detailed mechanisms through which p130Cas regulates these physiological processes is yet to be understood.

In this review we will focus on the role of p130Cas in breast cancer by first discussing its importance during normal mammary development and then its relevance in breast cancer.

## p130 CRK-Associated Substrate Expression in Normal Mammary Gland Development

Mammary gland development is a step process involving the activation of signaling pathways due to the coordinated stimulations of hormones, growth factors and extracellular matrix remodeling. It has been demonstrated that alteration either in the sequence of events or in the signaling pathways that governs mammary gland development can contribute to the onset and progression of breast cancer.

**FIGURE 1:**
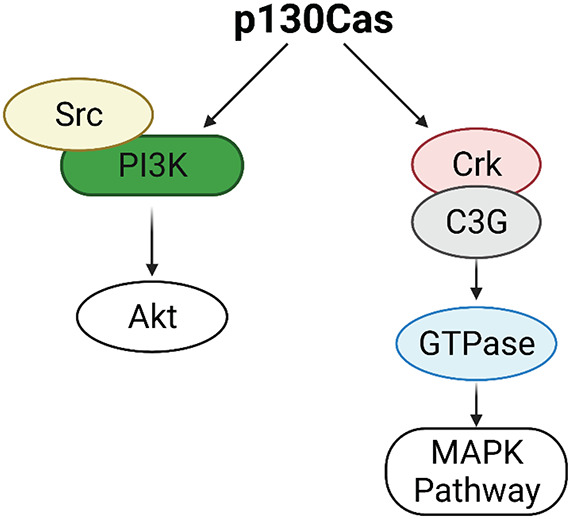
Activation of Erk1/2 MAPK and Akt pathways by p130Cas. p130Cas may activate AKT through the Src/PI3K axis. p130Cas association with Crk and C3G induces GTPases activation, leading to the upregulation of the MAPK pathway.

p130Cas/BCAR1 expression is detected in all the cell subtypes of the mouse mammary epithelium but it is highly enriched in the basal compartment. The mammary stroma shows very low levels of expression of p130Cas/BCAR1 (Tornillo et al., 2013). p130Cas/BCAR1 expression level is a crucial regulator of proper mammary development homeostasis. Indeed, transgenic mice overexpressing p130Cas/BCAR1 in the mammary gland show increased mammary branching morphogenesis *in vivo* during puberty, that results in extensive hyperplasia during pregnancy and lactation and delayed involution at the end of lactation. It has been shown that the morphological events occurring in presence of p130Cas overexpression are due to activation of proliferative signaling pathways involving Src, Erk1/2 MAPK, and Akt (Cabodi et al., 2006). The putative mechanism underlying Akt activation might be ascribed to the induction of the Src/PI3K axis, whereas Erk1/2 MAPKs are likely stimulated following Crk/C3G association with p130Cas and subsequent GTPases activation (Defilippi et al., 2006; Figure 1).

## Dysregulation of p130 CRK-Associated Substrate in Breast Cancer

### p130Cas/*BCAR1* and ER Positive Breast Cancer

**FIGURE 2:**
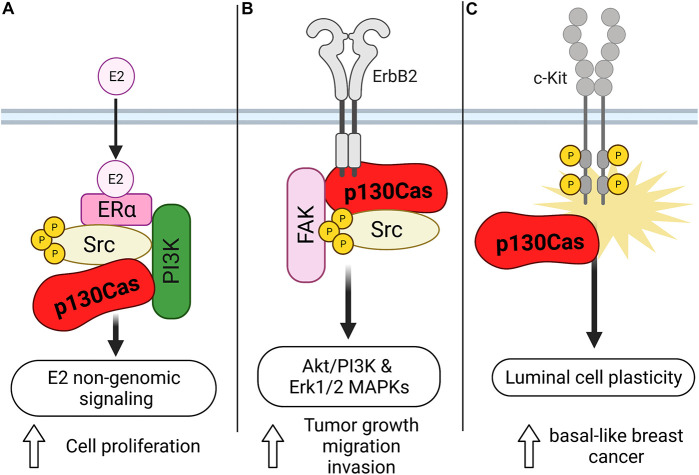
Schematic representation of the role of p130Cas in the different types of breast cancer. **(A)** Upon estrogen treatment, p130Cas promotes cell proliferation mediated by the non-transcriptional activity of ER in T47D cells. In this context, p130Cas forms a complex with ERa, Src, and the p85 subunit of PI3K. **(B)** p130Cas/ERBB2 direct interaction stimulates the Akt/PI3K and Erk1/2 MAPKs pathways, facilitating tumor growth, cell migration, and invasion. **(C)** p130Cap expression promotes the hyperactivation of c-Kit in mouse mammary epithelial cells, inducing luminal cell plasticity.

As mentioned before, the gene encoding p130Cas was identified by screening estrogen receptor-positive human breast cancer cells as responsible for promoting tamoxifen resistance and for this reason named *BCAR1* (Breast Cancer Antiestrogen Resistance 1). In human breast tumors, high levels of p130Cas expression correlates with a poor response to tamoxifen therapy, quicker disease recurrence and decreased patient survival (Dorssers et al., 2001), suggesting that p130Cas/*BCAR1* expression might be a convenient prognostic marker for patients affected by primary or metastatic breast cancer (van der Flier et al., 2000; Table 1). However, the mechanism through which p130Cas/*BCAR1* can promote resistance to tamoxifen, increased recurrence and poor patient survival was unknown. By using human estrogen receptor positive breast carcinoma T47D cells we came out with a model of the mechanism by which p130Cas regulates ER activity. We demonstrated that upon estrogen treatment, p130Cas rapidly and transiently associates with estrogen receptor alpha in a multimolecular complex containing Src kinase and the p85 subunit of PI3K (Figure 2A). Transient overexpression of p130Cas increases and accelerates estrogen-dependent Src kinase, Erk1/2–MAPKs activity and Cyclin D1 expression. Accordingly, p130Cas RNAi inhibits estrogen-dependent Erk1/2–MAPKs and Cyclin D1 induction, demonstrating that p130Cas is directly involved in the non-transcriptional activity of the ER, regulating estrogen-dependent cytosolic signaling pathways (Cabodi et al., 2004).

**TABLE 1:** Expression and prognostic value of p130Cas and p140Cas in breast cancer.

	Breast cancer subtype	Prognostic value	References
*p130Cas*	Invasive carcinomas	–	Cabodi et al., 2006
	ER and PR positive carcinomas	Poor relapse-free survival, poor overall survival, Tamoxifen resistance	van der Flier et al., 2000
	ERBB2-positive carcinoma	Poor overall survival, higher probability of developing a distant event	Cabodi et al., 2006; Tornillo et al., 2011
*p140Cap*	ERBB2-positive carcinoma	Lower probability of developing a distant event, increased survival	Grasso et al., 2017

### p130Cas/*BCAR1* and *ErbB2* Positive Breast Cancer

The ErbB2 positive breast cancer subtype represents around 20% of human breast cancer. The first hint of the implication of p130Cas in this aggressive subtype came by crossing transgenic animals overexpressing p130Cas in the mammary gland (MMTV-p130Cas) and those expressing the oncogenic form of the rat *Neu* gene, the homolog of the *ERBB2* human gene, called MMTV-NeuT. The resulting double-transgenic mice were characterized by accelerated onset of mammary tumor formation. Accordingly, *in vivo* and *in silico* analyses of human breast cancer confirmed that the amplification of *ERBB2* in combination with the overexpression of p130Cas induces a higher proliferation rate and an increased number of distant metastases, as well as a correlation with poor prognosis (Cabodi et al., 2006; Tornillo et al., 2011). To unravel the molecular mechanisms responsible for the synergic effect of p130Cas and ErbB2 in tumorigenesis, 3D cultures of MCF10A.B2 mammary epithelial cells were used. MCF10A.B2 cells express a chimeric and activatable ErbB2 receptor that can form spheroid structures called acini when grown in Matrigel (Muthuswamy et al., 2001), mirroring the architecture of the ductal lobular unit in the human mammary gland and representing a faithful model with which to study mammary gland biology *in vitro*. In this model, the concomitant activation of ErbB2 and overexpression of the p130Cas protein give rise to invasive protrusions. The invasive behavior induced by the synergism of p130Cas and ErbB2 results in the stimulation of Akt/PI3K and Erk1/2 MAPKs signaling pathways that in turn, lead to the activation of Rac1 GTPase and the secretion of the metalloproteinase MMP9 (Figure 2B; Cabodi et al., 2010; Tornillo et al., 2011). The molecular mechanisms by which p130Cas drive the 3D invasive phenotype are still not understood. It’s likely that its role as an adaptor protein is crucial in building a molecular hub very close to ErbB2. Indeed, in ErbB2-transformed cells, p130Cas is a crucial component of a functional molecular complex consisting of ErbB2, c-Src, and Fak. We demonstrated by using pharmacological inhibitors, that both MAPK and PI3K signaling cascades are required for the invasive behavior of p130Cas over-expressing and ErbB2 activated acini triggering invasion through distinct downstream effectors involving mTOR/p70S6K and Rac1 activation, respectively. It has also been demonstrated that Src activity seems to be dispensable, since treatment with the c-Src inhibitor SU6656 does not block cell invasion. The exact mechanisms through which p130Cas leads to activation of MAPK or PI3K are still not known. We can speculate that the activation of PI3K might be mediated by the interaction of p130Cas with the p85 subunit of PI3K, and the data with Src inhibitors suggest that at least Src is not involved in upstream regulation of PI3K.

In the p130Cas/ErbB2-mediated invasive process, the activation of MAPK and miR-23b downmodulation are instrumental to upregulate the transcriptional repressor Blimp1. Consistently, Blimp1 overexpression is detected in invasive breast cancer and correlates with metastatic status (Sciortino et al., 2017). Interestingly, the level of Blimp1 mRNA is upregulated in multi acinar structures of MCF10.B2 cells that result from p130Cas overexpression. In this experimental setting, Blimp1 expression is tightly controlled by the MAPK pathway, since treating the acini with MAPK inhibitor PD98059 dramatically reduces Blimp1 expression, leading to reduced formation of invasive protrusions. Cancer invasion represents a crucial event that allows tumor cells to disseminate in the stromal compartment by acquiring motile characteristics. Indeed, cancer cell invasiveness strictly depends on the possibility to undergo Epithelial-Mesenchymal Transition (EMT), a process during which cells lose adhesions to their neighbors and become more motile. It has been shown that p130Cas/*BCAR1* levels of expression impact on breast cancer cells EMT. As a matter of fact, in the highly invasive A17 mouse mammary tumor cells, p130Cas/*BCAR1* silencing induces loss of mesenchymal features and acquirement of epithelial-like traits, including the re-expression of the cell-cell adhesion molecule E-cadherin, thus affecting the EMT process involved in cancer progression. The mechanism through which p130Cas/BCAR1 expression induces the A17 cell invasive phenotype relies on its ability to increase the expression of cyclooxygenase-2 (Cox-2) (Bisaro et al., 2012). The p130Cas/Cox2-dependent EMT is effective both in the mouse and in the human setting. Indeed, while the role of Erk MAPK or PI3K/Akt has not been unraveled, c-Src and JNK kinases appear as sequential players in this axis and their pharmacological inhibition was sufficient to downregulate Cox-2 and to induce an epithelial phenotype. p130Cas is thus emerging as a critical player for onset and progression of many aggressive cancers, strengthening its relevance as an unfavorable prognostic marker and a putative therapeutic target, mostly in combination with high levels of ER, HER2, or Cox-2, respectively.

### p130Cas/*BCAR1* and Triple Negative Breast Cancer

In addition, it has been described that defective differentiation of mammary luminal progenitors predisposes to basal-like breast cancer (Molyneux et al., 2010). Indeed, the punctual analysis of p130Cas/MMTV transgenic mice, revealed that p130Cas overexpression occurs mainly in the luminal progenitor cell compartment and results in the expansion of luminal cells. The detailed characterization of the luminal cells showed that they aberrantly display basal cell features and reduced differentiation in response to lactogenic stimuli. Consistently, experiments performed in mouse mammary epithelial cells (MMECs) overexpressing p130Cas demonstrate that p130Cas expression leads to hyperactivation of the tyrosine kinase receptor c-Kit, indicating that high levels of p130Cas, via abnormal c-Kit activation, promote mammary luminal cell plasticity, thus providing the conditions for the development of basal-like breast cancer (Figure 2C; Tornillo et al., 2013). Accordingly, p130Cas is overexpressed in human triple-negative breast cancer (Tornillo et al., 2013; Table 1), implying that the increased expression of p130Cas may be a priming event for the onset of basal-like breast cancer.

## p130 CRK-Associated Substrate: Translational Applications

The signaling implicated in p130Cas/ErbB2-dependent tumorigenesis accounts for the direct interaction of p130Cas and ErbB2 (Cabodi et al., 2010; Tornillo et al., 2011). It was demonstrated that the direct binding of p130Cas to ErbB2 stabilizes ErbB2 protecting it from autophagy-mediated degradation by interfering with its ubiquitination. The increased stability of the receptor in presence of high p130Cas expression can be also responsible for resistance to Trastuzumab (Bisaro et al., 2016).

These data suggest important therapeutic and translational value of p130Cas in ErbB2 breast cancer and supports the hypothesis that p130Cas/ErbB2 interaction can serve as a potential target for the discovery and development of new anticancer agents, that can be used in combination with standard therapy to manage and control Trastuzumab resistance.

Recently, two potential inhibitors of p130Cas/ErbB2 interaction were identified by structure-based virtual screening. Their experimental validation was performed *in vitro* and in ErbB2-positive breast cancer cellular models. The results highlight that both compounds interfere with p130Cas/ErbB2 binding and significantly affect cell proliferation and sensitivity to Trastuzumab (Costamagna et al., 2019). This study supports p130Cas/ErbB2 complex as a potential breast cancer target and shows the druggability of this protein-protein interaction (PPI) that might benefit from a more advanced optimization effort for therapeutic applications.

Compared with other breast cancer subtypes, TNBC is highly invasive, has a high early recurrence rate and is exceptionally difficult to treat. The lack of ER, PR, and ErbB2 expression renders the tumor unresponsive to hormonal therapies or ErbB2-targeted therapies. Therefore, development of new TNBC treatment strategies has become an urgent clinical need (Yin et al., 2020). It has been demonstrated that TNBCs express high levels of p130Cas, that promote mammary luminal cell plasticity, thus providing the conditions for the development of basal-like breast cancer (Tornillo et al., 2013). It is possible to envision an application for protein-protein interaction inhibitors against p130Cas also in this context. Previous data suggest that p130Cas mediates its role in TNBC through increased c-Kit activation, therefore it would be reasonable to validate p130Cas/c-Kit direct interaction and then start a drug discovery screening to identify potential p130Cas/c-Kit interaction inhibitors.

## Identification of p140Cap (p130Cas-Associated Protein) and Its Main Characteristics

As previously described, p130Cas is able to interact with different molecules, involved in several signaling processes, in particular cell adhesion, motility, and transformation (O’Neill et al., 2000; Bouton et al., 2001). In order to identify and characterize new p130Cas interactors, an affinity chromatography experiment was performed in human cells. Among others, a 140 kDa protein was detected, named p140Cap for p130Cas-associated protein, also known as SNIP, as Snap25 Interacting Protein in rat brain (Chin et al., 2000). p140Cap is an adaptor protein that indirectly associates with p130Cas and it has been shown to be involved in integrin- and Epidermal Growth Factor (EGF)-dependent signaling (Di Stefano et al., 2004). It is encoded by the *SRCIN1* gene, located on chromosome 17q12. One of its main functions is the binding and activation of C-terminal Src kinase (CSK) leading to Src inhibition, as well the downstream signaling with the related tumor properties (Di Stefano et al., 2007). In particular, 140Cap stabilizes adherens junctions and inhibits EGF Receptor (EGFR) and Ras signaling through the dual control of both Src and Ras activities, thus affecting crucial tumor features such as growth and invasion (Damiano et al., 2010).

While p130Cs is ubiquitously expressed, p140Cap expression is highly tissue-specific. Indeed, analyzing several tissues and cellular lines (Di Stefano et al., 2004), it was apparent that the p140Cap protein is physiologically expressed in neural and epithelial tissues and in a significant subset of cancers including breast cancer and neuroblastoma (Salemme et al., 2021). Interestingly, in both these types of cancers p140Cap has been demonstrated to act as tumor suppressor protein, impairing cancer properties and correlating with a better prognosis (Di Stefano et al., 2007; Grasso et al., 2017, 2020). In addition to Src inhibition, p140Cap has been shown to affect Rac1 GTPase activity and to decrease Tiam1 activation in breast cancer cells (Grasso et al., 2017; Chapelle et al., 2020).

## p140Cap Expression in Mammary Gland and Its Role in Breast Cancer

In physiological conditions, the p140Cap protein is detectable in the human mammary gland, where it is specifically expressed in alveolar luminal cells. A first immunohistochemical analysis with specific monoclonal antibodies on a small cohort of human breast cancers revealed that p140Cap is lost in the most aggressive and highly proliferative cancers, suggesting an inverse correlation between its expression and the state of malignancy (Damiano et al., 2010). However, in order to assess a prognostic relevance for p140Cap expression, a second study was performed. In particular, a cohort of 622 breast cancer patients in tissue microarray was analyzed. The results revealed that a p140Cap positive status was associated with negative lymph node status, ER and progesterone receptor (PgR)-positive status, small tumor size, low grade, low Ki67 status, and correlates with a better prognosis in ERBB2-positive patients.

Interestingly, it emerged that the p140Cap coding gene *SRCIN1* could be included in the *ERBB2* amplicon, being the *SRCIN1* gene located on human chromosome 17q12, one million base pairs centromeric to the *ERBB2* gene. In fact, the analysis of 200 *ERBB2*-amplified tumors showed that the *SRCIN1* gene is often (55–60% of *ERBB2*-amplified breast cancer patients), but not necessarily co-amplified with *ERBB2*. The prognostic effect of p140Cap was finally underlined in this subgroup of *ERBB2*-amplified breast cancer patients, where the Kaplan–Meier analysis of these tumors evidenced that *SRCIN1* amplification is associated with a significant improved survival and an high expression of p140Cap predict a lower probability of developing metastasis.

Taken together these data suggest a relevant role for p140Cap as prognostic marker in *ERBB2*-amplified breast cancer patients, highlighting its tumor suppressor functions in this breast cancer subtype (Grasso et al., 2017).

## p140Cap and Signaling

### Src and Csk Phosphorylation

**FIGURE 3:**
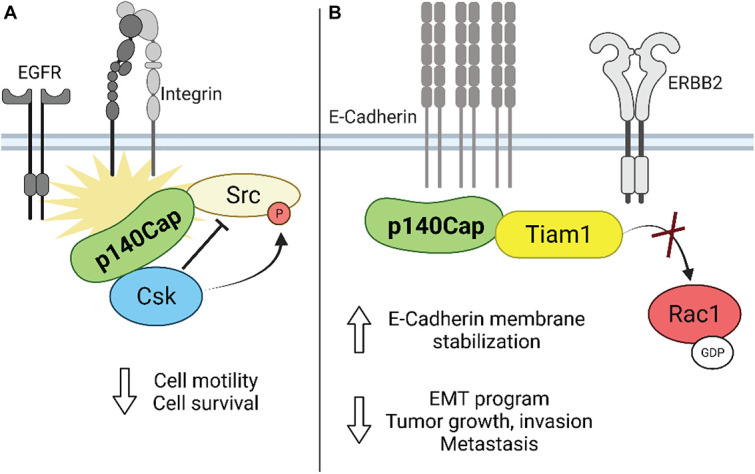
Schematic representation of the role of p140Cap in breast cancer. **(A)** Upon integrin and growth factor-mediated signaling, p140Cap inhibits Src activity by binding to the Src inhibitor Csk. **(B)** p140Cap counteracts the EMT process by increasing E-cadherin stability at the plasma membrane in luminal A cell line MCF7. Moreover, p140Cap downregulates Rac1 activation by interfering with the Rac1-GEF Tiam in ERBB2 transformed cells.

As reported above, upon integrin-mediated adhesion, Src kinase regulates cell growth, spreading and migration through increased phosphorylation of Fak as well as of other key adaptor molecules, like p130Cas (Mitra et al., 2005; Defilippi et al., 2006). Interestingly, by loss- and gain-of-function approaches in *in vitro* models of breast cancer, it was found that p140Cap affects breast cancer cell motility and invasion. Indeed, high levels of p140Cap result in inhibition of integrin- and EGFR-dependent Src activation, through its ability to directly associate with it (Di Stefano et al., 2007; Damiano et al., 2010). Mechanistically, it was found that p140Cap behaves as a novel binding partner in the cell machinery recruiting Csk and Src, through the binding of Csk on p140Cap with the tyrosines inserted in EGLYA/EPLYA motif and of Src with the proline rich domains, respectively, regulating in this way their activity and downstream signaling in breast cancer cells (Figure 3A; Di Stefano et al., 2007; Repetto et al., 2013). Effectively, the direct mutagenesis of the tyrosine present in the EPLYA/EGLYA peptides, is sufficient to disrupt the ability of p140Cap to bind Csk and thus to inhibit Src activity.

### p140Cap and Epithelial-Mesenchymal Transition

As shown before, in the *ERBB2*-amplified subgroup of breast cancer patients, p140Cap expression predicts a significantly lower probability of developing distant events (Grasso et al., 2017). EMT is the first step in the metastatic process, increasing the migratory ability of cancer cells. Effectively, in primary epithelial cancer cells derived from NeuT and p140-NeuT tumors in the BALB/c background, the presence of p140Cap correlates with the up-regulation of both E-cadherin mRNA and cell surface protein levels (Grasso et al., 2017). Furthermore, p140Cap exerts an overall inhibitory effect on counteracting the EMT invasive program of ERBB2 tumors, as shown by a marked down-regulation of the EMT transcription factors Snail, Slug, and Zeb1 as well as by the reduction of the mesenchymal cell–cell adhesion protein N-cadherin. Noteworthy, p140Cap is also able to interact with E-Cadherin, both with classical biochemical approaches (Damiano et al., 2010) or by a comprehensive analysis of the p140Cap interactome in breast cancer cells (Chapelle et al., 2019). Overall, these data indicate its possible contribution in strengthening the adherence junction stability through the relocalization and immobilization of E-cadherin at the plasma membrane (Figure 3B). Future studies will determine how p140Cap participates in these molecular interactions in a spatial and temporal manner and how it affects E-cadherin junction stability, which is a key step in counteracting the EMT process and more in general the tumor progression.

### p140Cap Affects Tiam1-Dependent Rac1 Migration

The clinical evidence that p140Cap correlates with a favorable outcome in ERBB2 breast cancer patients suggest that p140Cap is able to curb the intrinsic biological aggressiveness of ERBB2 tumor (Grasso et al., 2017). Indeed, p140Cap confers to ERBB2 transformed cells both limited *in vivo* tumor growth ability and impaired spontaneous lung metastasis formation. This less aggressive phenotype is likely linked to reduced cell proliferation, assessed by a decreased staining of the proliferative marker PCNA in tumors, increased sensitivity to apoptosis, and strong inhibition in the EMT program observed in p140Cap expressing tumor cells. As reported above, p140Cap limits the integrin and the EGFR signaling pathways by activating Csk and inhibiting Src activity, thus leading to impaired cell spreading, motility, and invasion (Di Stefano et al., 2007). In the same context, p140Cap limiting integrin or growth factor signalings, is able to counteract tumor features such as cell migration and invasion. On the same line, Grasso et al. by using SKBR3 breast cancer cells as a model of ERBB2 amplification, reported that in a transwell assay, migration was significantly decreased in p140Cap-overexpressing cells as well as increased in MDA-MB-453 p140Cap-silenced cells, an additional model of *ERBB2* gene amplification.

Unexpectedly, both in NeuT and in SKBR3 cells, p140Cap expression did not affect the activation of the Src kinase and the phosphorylation of its effectors such as p130Cas and paxillin, suggesting that in ERBB2 transformed cells p140Cap controls cell migration in an additional way. In presence of p140Cap the Rac1 GTPase activity was significantly decreased as reported by the *in vitro* pull-down of active Rac1. Concomitantly, the decreased Rac1 activity in p140Cap expressing cells was dependent on decreased activity of a specific Rac1-GEF, namely Tiam1, as found in a GST-RacG15A *in vitro* pull-down assay. The same results were also obtained in p140Cap-silenced MDA-MB-453 cells. Moreover, p140Cap and Tiam1 co-immunoprecipitated, indicating that p140Cap associates in a molecular complex with Tiam1, and suggesting that this interaction may be responsible for reducing Tiam1 activity (Grasso et al., 2017). As a consequence of impaired motility, p140Cap expression in ERBB2 preclinical models is also able to reduce the number of metastases in *in vivo* assays of spontaneous and experimental metastasization (Figure 3B).

## Transcriptional and Posttranscriptional Control of p130 CRK-Associated Substrate and p140Cap Expression

The mechanisms underlying the transcriptional regulation of p130Cas and p140Cap in physiology and pathology are still poorly investigated. The transcriptional control of p130Cas/*BCAR1* in human breast cancer cells is partly due to the transcription factor EGR1 and its coregulator NAB2 (Kumbrink and Kirsch, 2012). Interestingly, p130Cas signaling mediates the induction of both EGR1 and NAB2, which, in turn, up-regulates p130Cas expression in a positive feedback loop.

To date, we are not aware of any transcription factors involved in the regulation of *SRCIN1* expression. The identified mechanisms responsible for its deregulation in cancer are mainly related to chromosome rearrangements and microRNA control. Besides the co-amplification with *ERBB2* in breast cancer, the *SRCIN1* gene might be lost or disrupted in some cases of aggressive neuroblastoma due to the 17q12 chromosomal rearrangement (Grasso et al., 2020). Although the clinical relevance of *SRCIN1* expression in neuroblastoma has been demonstrated, since it is an independent risk factor inversely correlated to disease aggressiveness, the importance of these genetic aberrations should be better investigated in a larger cohort of patients. Recently, Damez-Werno et al. (2016) discovered that the *SRCIN1* gene is transcriptionally activated in the nucleus accumbens (NAc) of mice brains following cocaine exposure thanks to a particular chromatin modification. In this specific brain area, *SRCIN1* exhibited reduced asymmetric dimethylation of R2 on the histone H3 (H3R2me2a), leading to consequent *SRCIN1* induction in the NAc, decreased Src signaling, and reduction of the rewarding effects of cocaine, including self-administration of the drug. It is crucial to identify the transcriptional and epigenetic mechanisms determining p140Cap and p130Cas deregulation in cancer to develop new therapeutic strategies.

Several microRNAs (miRNAs) affect the expression of these adaptor proteins. The post-transcriptional control of p140Cap by miRNAs occurs in different types of cancer and involves many miRNAs, as extensively discussed elsewhere (Salemme et al., 2021). The only miRNA that acts as a negative regulator of p140Cap in breast cancer is miR-150, which promotes cell migration, invasion, and expression of EMT markers in breast cancer cells. Three miRNAs, namely miR-24-3p, miR-362-3p, and miR-329, are known to direct target p130Cas and inhibit its protein levels, leading to impaired breast cancer cell migration and invasion (Kang et al., 2016, 2017).

## Targetable Pathways in Breast Cancer From p140Cap and p130Cas Associated Protein Interactome Studies

Given the presence of multiple protein-binding modules, adaptor proteins can facilitate the assembly of signaling complexes regulating cellular signals both spatially and temporally (Flynn, 2001). Understanding the nature and the functional role of the multi-protein complexes associated with p140Cap and p130Cas adaptor proteins might be critical to discover new biologically relevant and potentially targetable pathways in the context of breast cancer.

Recently, we generated a p140Cap interactome from the ERBB2-positive breast cancer TuBo cell, a clonal line established *in vitro* from a BALB-NeuT mouse mammary carcinoma (Rovero et al., 2000; Chapelle et al., 2019). The identification and functional characterization of p140Cap co-immunoprecipitated proteins, performed by Mass Spectrometry and subsequent bioinformatic analysis, revealed 374 putative interacting partners. Among these proteins are present crucial components of signaling pathways that regulate specific cellular functions such as cell-substrate junction, focal adhesion organization, cell-cell adhesions, cell cycle and apoptosis, and protein homeostasis (Chapelle et al., 2019).

One putative interactor is E-cadherin, which was already demonstrated to co-immunoprecipitate with p140Cap in the luminal A MCF-7 cells, where E-Cadherin is immobilized by p140Cap at the cell surface, strengthening adhesion between cells and curbing breast cancer cell migration (Damiano et al., 2010). Members of the catenin family, such as α-, β-, and δ catenin, were also identified as interactors, emphasizing the role of p140Cap in the context of the cadherin/catenin-based adhesion system in breast cancer cells. Besides the well-characterized roles in cell adhesion, β-catenin acts as a transcriptional co-activator in the canonical Wnt signaling (Clevers and Nusse, 2012), raising the hypothesis that p140Cap might participate in the regulation of the Wnt signaling pathway in breast cancer.

Analysis of the functional relationships between proteins within the interactome highlighted the presence of defined protein clusters characterized by specific functions. One protein cluster was enriched with kinase domain-containing proteins, such as Src, ERBB2, and ERBB2IP (ERBIN), reinforcing the idea that p140Cap can associate and regulate kinases implicated in breast cancer transformation and progression. The interactome also contains proteins involved in actin cytoskeleton remodeling, including Actinin B and Actin Remodeling Protein (FLII), several F-actin capping proteins, the motor protein Myosin 6 (MYO6) and Flotillin, which localizes to the caveolae and plays a role in vesicle trafficking. To better understand the function of these interactors in the tumor-suppressing roles of p140Cap, it’s mandatory to address their biological role in an experimental breast cancer context and if this role is somewhat compromised by the interaction with p140Cap.

Although less is known on the global p130Cap interactome in breast cancer models, Evans et al. (2017) identified the interactome of p130Cas in human endothelial cells in response to VEGF treatment. They reported that VEGF stimulation induces an enrichment in p130Cas interactors implicated in cell motility, actin cytoskeletal dynamics, and angiogenesis, such as IQGAP, Profilin-1, FLII, MYO6, and MRCKβ. It is reasonable to hypothesize that some of these interactors might be directly or indirectly associated with p130Cas independent from the cellular context. Interestingly, FLII, MYO6, and IQGAP are interactors shared by both adaptor proteins, indicating that p140Cap and p130Cas might regulate common signaling pathways involved in cytoskeletal remodeling by interacting with similar molecular complexes. However, it would be of great interest to investigate the p130Cas interactome in the breast cancer TUBO cells, which will allow a better understanding of the opposing contributions of p140Cap and p130Cas adaptor proteins in controlling cancer cell signaling pathways (Cabodi et al., 2006).

## Conclusion

**FIGURE 4:**
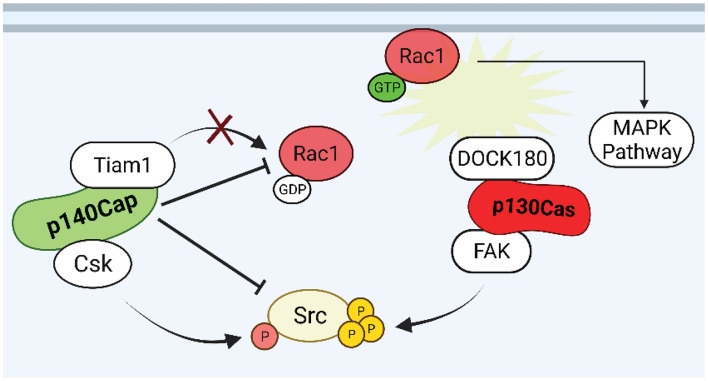
Putative p130Cas and p140Cap points of crosstalk in breast cancer. Rac1 and Src are the possible points of crosstalk in which p130Cas and p140Cap may exert their antagonistic functions in the regulation of tumorigenic signaling. While Rac1 GTPase is kept inactive by p140Cap through the inhibition of the GEF Tiam1, the assembly of p130Cas/DOCK180 complex induces the recruitment of active Rac1 to the cell membrane. The p140Cap/CSK and p130Cas/FAK interactions contribute to the inhibition and activation of Src activity, respectively.

The above findings provide evidence for a role of p130Cas as a positive regulator of both proliferation and survival in normal and transformed mammary epithelial cells. In contrast, based on the collected data, p140Cap, in the same breast cancer cell models, behaves as an oncosuppressor which opposes and interferes with cancer features. Several molecular pathways have already been depicted through which p130Cas and p140Cap may exert their opposite properties in breast cancer (Figure 4). The fact that the two proteins have common interacting partners, such as the Src kinase, with opposite effects, further indicate that the two proteins share a common network and that a temporal analysis of their reciprocal localization and kinetics of interactions with specific interactors would be of great interest to shed light on their functions. The comprehensive analysis of p140Cap interactome in BC cells has recently provided data on its involvement in several additional biological networks relevant for cancer progression. The analysis of the p130Cas interactome in the same model would provide additional knowledge to the comprehension of this complex network. Moreover, we have recently generated genetically modified mice in which either p130Cas or p140Cap can be specifically ablated in a specific tissue (Del Pilar Camacho Leal et al., 2018; Russo et al., 2019). These models could be exploited in the mammary gland to further address the impact of each protein in physiological development or in tumor progression, focusing on the functional balance of the common network of interacting proteins.

Finally, further analysis is needed of both *BCAR1* and *SRCIN1* gene status and regulation in specific breast cancer subtypes, in order to address their real contribution to the biological heterogeneity of human tumors in terms of patient stratification. On the other hand, since many tumors express p130Cas, but do not express relevant levels of p140Cap, the existing knowledge on miRNAs acting on p130Cas and p140Cap expression in human tumors, will provide the testable hypothesis on the use of specific anti-miRNAs to enhance an appropriate tumor response in pre-clinical models. Overall, the dissection of p130Cas and p140Cap biological features and their regulated pathways in breast cancer highlight the potential clinical impact of their reciprocal expression on patient stratification, as being relevant key players for patient outcome.

From the ancient Chinese philosophy of the yin and yang (bright-black or positive-negative) concept of dualism, the detailed study of p130Cas and p140Cap proteins suggests that in normal tissues they can be not only opposite or contrary forces, but actually be complementary, interconnected, and interdependent in the natural world. According to this philosophy, everything has both yin and yang aspects (for instance, shadow cannot exist without light). Additional studies are needed to better address if a similar concept can be applied to the p130Cas/p140Cap network (Box 1).

**Critical unanswered questions on p130CAS and p140CAP in breast cancer.**

Key Unanswered Questions

• How is BCAR1/p130Cas transcriptionally regulated and which are the mechanisms responsible for its overexpression In breast cancer?
• Despite the already identified p130Cas interactors, which is the comprehensive p130Cas interactome in breast cancer cells?
• Which is the prognostic value of p140Cap in the other subtypes of breast cancer, except for the ErbB2-amplified tumors?
• Are there any point of cross-talk between p130Cas and p140Cap in breast cancer?
• Are the multi-protein complexes associated with p130Cas and p140Cap critical to uncover new biologically relevant and potentially targetable pathways in the context of breast cancer?

## Author Contributions

GC, VS, and PD conceived and wrote the part relating to p140Cap/SRCIN1. DN, AC, and SC conceived and wrote the part relating to p130Cas/BCAR1. SC and PD revised the manuscript and the figures. All authors contributed to the article and approved the submitted version.

## Conflict of Interest

The authors declare that the research was conducted in the absence of any commercial or financial relationships that could be construed as a potential conflict of interest.

## Publisher’s Note

All claims expressed in this article are solely those of the authors and do not necessarily represent those of their affiliated organizations, or those of the publisher, the editors and the reviewers. Any product that may be evaluated in this article, or claim that may be made by its manufacturer, is not guaranteed or endorsed by the publisher.
